# DNA Unwinding Driven by Gold Nanoparticles

**DOI:** 10.3390/nano15241872

**Published:** 2025-12-13

**Authors:** Liat Katrivas, Galina M. Proshkina, Sergey M. Deyev, Alexander B. Kotlyar

**Affiliations:** 1 The George S. Wise Faculty of Life Sciences, University Center for Nanoscience and Nanotechnology, Tel Aviv University, Tel Aviv 6997801, Israel; liatkatrivas@mail.tau.ac.il; 2 Shemyakin-Ovchinnikov Institute of Bioorganic Chemistry, Russian Academy of Science, 16/10 Miklukho-Maklaya Street, Moscow 117997, Russia; gmb@ibch.ru (G.M.P.); deyev@ibch.ru (S.M.D.)

**Keywords:** DNA unwinding, gold nanoparticles, AFM, DNA nanostructures

## Abstract

We demonstrate that gold nanoparticles (AuNPs) are capable of unwinding double-stranded (ds) DNA. Upon unwinding, the exposed nucleobases of the separated strands adsorb onto the nanoparticle surface, resulting in the coating of the particles. The unwinding process was characterized by Atomic Force Microscopy (AFM) and absorption spectroscopy. Our results show that AuNPs initially bind to single-stranded overhangs at the duplex termini, forming dsDNA–nanoparticle dumbbells. This binding event subsequently initiates the separation of the DNA strands. As the unwinding proceeds, the nanoparticles become progressively wrapped by the unwound DNA strands, which leads to a gradual reduction in the interparticle distance within the dumbbells. This process is driven by the strong affinity of nucleobases for the gold surface. The efficiency of DNA unwinding was found to depend strongly on both nanoparticle size and temperature. These findings provide new insights into DNA-nanoparticle interactions and may facilitate the rational design of DNA–AuNP hybrid nanostructures such as dumbbell-shaped conjugates for applications in DNA-based nanoelectronics, biosensing, and self-assembled nanomaterials.

## 1. Introduction

Gold nanoparticles (AuNPs) are among the most extensively studied nanomaterials due to their unique optical and electronic properties, biocompatibility, and facile synthesis. Their strong affinity for biomolecules has enabled the development of hybrid nanostructures with applications in biosensing, plasmonics, and molecular electronics (for reviews see [1,2]). Among these hybrids, DNA-nanoparticle conjugates are particularly attractive due to the programmable nature of DNA and the unique optical and electronic properties of AuNPs [3,4,5,6]. DNA has been shown to be particularly effective as a molecular scaffold for organizing AuNPs with nanometer precision, leading to constructs such as AuNP-DNA dumbbells in which two nanoparticles are bridged by a dsDNA linker [7,8,9,10]. Such architectures, as well as more complex ones [11,12,13], have been exploited for studying charge transport through DNA [14], plasmonic coupling [7,15,16], and single-molecule enzymatic processes [17,18]. The most traditional and common approach to conjugating DNA to AuNP relies on thiol-modified oligonucleotides: DNA bearing SH-groups which bind to the gold NP surface [19,20]. An alternative approach utilizes the intrinsic binding of AuNPs to nucleic bases. Gold nanoparticles are known to tightly bind to the DNA nucleobases, leading to the formation of stable conjugates with single-stranded (ss) DNA [21]. Nitrogen atoms play a central role in this interaction, with binding affinities in the following order: adenine > guanine > cytosine > thymine [21,22]. Since in a dsDNA the bases are hybridized and hidden within the helical core, they are inaccessible to the AuNP surface, limiting binding primarily to ssDNA regions and fragments.

Importantly, the ability of AuNPs to bind DNA strongly depends on the nature of their surface ligands. Tightly binding ligands, such as thiols or BSPP (bis(p-sulfonatophenyl)phenylphosphine), prevent the adsorption of weaker ligands such as nucleic bases or proteins. For example, BSPP-coated AuNPs do not interact with unmodified DNA bases but readily form conjugates with thiolated oligonucleotides, a property that has been widely exploited in the construction of nanoparticle–DNA nanostructures [20]. In contrast, citrate-protected AuNPs are capped by weakly bound citrate ions, which can be readily displaced by nucleic bases, thiols, or amines. Consequently, citrate-protected nanoparticles exhibit strong interactions with DNA bases [23]. Citrate-capped AuNPs (Cit-AuNPs) therefore can selectively bind to ss regions such as overhangs or nicks, effectively discriminating between ss and ds segments [23,24,25]. This property enables nanoparticles to bind to specific sites and can induce local unwinding of the DNA duplex when interacting with exposed ss ends [26,27]. This property allows nanoparticles to be specifically positioned at ssDNA regions, triggering local unwinding of the DNA duplex [26,27]. The weak citrate capping is key to this behavior, as citrate can be displaced by the nucleobases, allowing a strong base–Au interactions.

This study demonstrates that Cit-AuNPs can selectively bind ss DNA overhangs and unwind ds DNA segments of tens to hundreds of base pairs. This ‘helicase-like’ activity of the particles is driven by strong interactions between the nucleobases of the unwound DNA strands and the particle surface. The process described here offers an alternative to thiolated oligonucleotides for constructing functional conjugates for applications in nanoelectronics, plasmonics, and single-molecule biosensing.

## 2. Materials and Methods

### 2.1. Materials

Unless otherwise stated, the reagents were obtained from Sigma-Aldrich (St. Louis, MO, USA) and were used without further purification. Endonucleases (EcoRI, SmaI, PluTI, AflIII) and pUC19 plasmids were purchased from New England Biolabs (NEB) (Ipswich, MA, USA). Oligonucleotides were purchased from Integrated DNA Technologies, Inc. (IDT, Coralville, IA, USA).

### 2.2. Methods

#### 2.2.1. The 8 nm AuNP Synthesis

Spherical citrate-capped 8 nm AuNPs were obtained by enlarging 5 nm seeds via surface reduction of gold ions. The seeds were prepared essentially as described in [28]. Briefly, to 100 mL of 0.29 mM HAuCl_4_ and 0.5 mM sodium citrate mixture, 1 mL of freshly prepared 0.2 M NaBH_4_ solution was added under vigorous stirring. The wine-red solution was left overnight at ambient temperature. Further enlargement of the 5 nm seeds was conducted as follows: 100 mL of 0.22 mM HAuCl_4_ in DDW was heated to boiling in a round neck glass flask. Approximately 1 mL of 0.1 M potassium-citrate solution (pH 6, Cit-K), and 10 mL of 5 nm particle seeds were added simultaneously into the boiling solution under vigorous stirring and left to stir for another 5 min in the bath. The flask was then pulled out from the glycerol bath and left to slowly cool to 25 °C. 5 mL of 0.1 M Cit-K solution was added to the particle solution drop-by-drop under vigorous stirring. The particles were then concentrated using 50 K Amicon Ultra-filtration (Merck KGaA, Darmstadt, Germany) 15 mL centrifugal units on a benchtop Eppendorf centrifuge at 1000 rpm for 15 min at 20 °C. Particle concentration was determined by measuring absorbance at 525 nm using molar extinction coefficient of 5 × 10^7^ M^−1^ cm^−1^.

#### 2.2.2. The 15 nm AuNP Synthesis

Spherical citrate-capped 15 nm AuNPs were synthesized via citrate-mediated reduction of HAuCl_4_, following a modified Turkevich method [29]. Briefly, 200 mL of 0.22 mM HAuCl_4_ solution was placed in a 500 mL Erlenmeyer flask. The solution was transferred to a glycerol bath preheated to 140 °C. Once the solution reached boiling, 3 mL of 34 mM trisodium citrate (filtered through an Amicon Ultra Centrifugal Filter, 3 kDa MWCO) was quickly added under vigorous stirring. The reaction was considered complete when the color of the suspension no longer changed, typically after 7 min. The flask was then removed from the bath and left to slowly cool to 20 °C. The resulting AuNP suspension remained stable for several months under ambient conditions. The particles were concentrated using 100 K Amicon Ultra-filtration 15 mL centrifugal units on a benchtop Eppendorf centrifuge for 15 min at 1500 rpm and 20 °C. Particle concentration was determined by measuring absorbance at 527 nm using molar extinction coefficient of 3 × 10^8^ M^−1^ cm^−1^.

#### 2.2.3. Cleavage and Purification of pUC19 Plasmid

Circular pUC19 plasmid was linearized using EcoRI, SmaI, PluTI, AflIII endonucleases according to standard company protocol. Following cleavage, the mixture containing: DNA, enzyme(s), and buffer components was chromatographed on a 1.5 × 5 cm (10 mL volume) Sepharose CL-2B column equilibrated with 5 mM Cit-K buffer (pH 6). The void volume fractions containing DNA, free of enzyme and buffer components were collected. Concentration of plasmids (in strands) was determined by measuring absorbance at 260 nm using molar extinction coefficient of 4 × 10^7^ M^−1^ cm^−1^.

#### 2.2.4. Incubation of DNA with AuNPs

DNA eluted from the column (see above) was concentrated using 10 K, 0.5 mL Amicon centrifugal units at 10,000 rpm for 10 min at ambient temperature. The concentrated solution was then mixed with 8 nm or 15 nm AuNPs at varying molar ratios in 5 mM Cit-K buffer (pH 6.0) at 4 °C (unless mentioned otherwise). Both DNA and AuNPs were cooled prior to mixing. Incubation times were varied as specified in each individual experiment. Following incubation, 1 mM BSPP was added to the mixture.

#### 2.2.5. Estimation of the Number of Bases Capable of Binding to a 15 nm Gold Nanoparticle

15 mL of 15 nm AuNPs (OD at 525 nm = 1) were placed in a 25 mL glass beaker. 30 µL of a 40-base ssDNA (5′-CACCGCGTACTAATAGCACAATTGTCGTGGTGACGCTCGG-3′) solution (OD at 260 nm = 130) was added to the stirred AuNP suspension and incubated for 1 h at ambient temperature. Then, 150 µL of 3 M KCl was added dropwise over 15 min under vigorous stirring.

The suspension was then left for 3 h at ambient temperature. The particles were centrifuged in 15 mL Amicon Ultra 100 kDa centrifugal filter units at 2000 rpm for 5 min at 20 °C. The filtrate was discarded, and the particle-containing retentate was resuspended in 15 mL of deionized water (DDW). The sample was centrifuged a second time under identical conditions. The retentate fraction containing the particles was transferred to a 15 mL Eppendorf tube and centrifuged at 14,000 rpm for 10 min on a benchtop Eppendorf centrifuge. The pellet was resuspended in ~10 µL of DDW. Approximately 1–2 µL of the nanoparticles were diluted in 1 mL of DDW in a 1 cm cuvette. The particles were dissolved by incubation with 10 mM KCN for 30 min. The spectrum of the CN-dissolved nanoparticles was compared to that of the control AuNPs (not incubated with the oligonucleotide). The oligonucleotide concentration in the conjugate was calculated from the spectral analysis using extinction coefficient of 3.8 × 10^5^ M^−1^ cm^−1^ at 260 nm. Concentration of 15 nm AuNPs particles was calculated using extinction coefficient of 3 × 10^8^ M^−1^ cm^−1^ at 527 nm.

#### 2.2.6. Absorption Spectroscopy

UV-Vis absorption spectra were recorded under ambient conditions using a Scinco S-3100 spectrophotometer (Seoul, Republic of Korea).

#### 2.2.7. Gel Electrophoresis

Samples (~20 µL per lane) were loaded onto 2.5% (unless mentioned otherwise) agarose gel (7 × 7 cm^2^) and electrophoresed for 1 h at 4 °C and 100 V. Tris-acetate-ethylenediaminetetraacetic acid (TAE) in addition to being used to prepare the agarose, also served as the running buffer.

Discrete red-colored DNA–particle conjugates were cut from the gel using a razor blade and transferred into a dialysis bag (MWCO 14000) containing TAE buffer. The eluted conjugates were then concentrated using 10 K, 0.5 mL Amicon filtration units at 10,000 rpm for 10 min at ambient temperature. The concentrated conjugates were subsequently imaged by AFM.

#### 2.2.8. AFM

A total of 5 μL of the concentrated conjugates (absorbance at 260 nm ≈ 100 mAU) was diluted into 100 μL of 1 mM Mg-Acetate and deposited on freshly cleaved mica for 5 min under ambient conditions. The surface was subsequently rinsed with DDW and quickly dried by a stream of nitrogen. AFM imaging was performed on a Solver PRO AFM system by NT-MDT (Moscow, Russia), in a semi-contact mode, using 130 μm long Si-gold-coated cantilevers by ‘ScanSens’ (Hamburg, Germany) with a resonance frequency of 70–180 kHz. The images were “flattened” (each line of the image was fitted to a second-order polynomial, and the polynomial was then subtracted from the image line) with Nova image processing software version 3.5 (by NT-MDT-, Moscow, Russia) and WSxM software [30].

## 3. Results and Discussion

### 3.1. Binding of AuNPs to DNA

**Figure 1:**
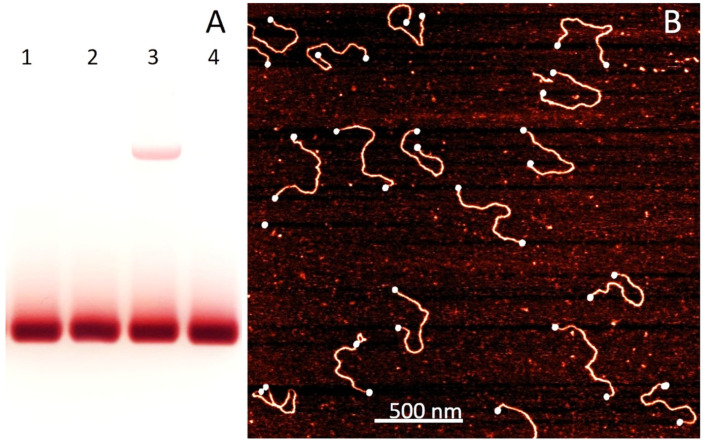
Electrophoresis and AFM analysis of nanoparticle–DNA conjugates. (**A**)—Electrophoresis of 8 nm AuNPs (lane 1) incubated for 30 min at 4 °C with circular pUC19 (lane 2), EcoRI-linearized pUC19 (lane 3) bearing ss overhangs at both DNA ends, or blunt-ended SmaI-linearized pUC19 (lane 4). Samples were loaded onto a 2% agarose gel and ran for 50 min at 100 V under ambient conditions. (**B**)—AFM imaging of the conjugate formed between EcoRI-linearized pUC19 and 8 nm AuNPs. The conjugate, electroeluted from the upper band gel area in lane 3, was imaged by AFM as described in Section 2. AuNPs appear as bright dots at the DNA termini. The addition of 1 mM BSPP to the incubation mixture completely abolished conjugate formation with DNA bearing single-stranded overhangs.

We first examined the specific binding of citrate-stabilized AuNPs to ss overhangs at the termini of kilobase-long ds DNA molecules. When 8 nm particles were incubated with EcoRI-linearized pUC19 in 5 mM Cit-K buffer (pH 6), conjugates were obtained in which two AuNPs were specifically bound to the opposite ends of the DNA duplex. Agarose gel electrophoresis enabled complete separation of the conjugate from excess AuNPs (Figure 1A lane 3, Figure 1B). The conjugate migrated much slower than the free particles (intense lower band in Figure 1A, lanes 1–4) and appears as a distinct upper band in the gel (Figure 1A, lane 3). The reduced mobility of the conjugate compared to free AuNPs is due to its larger size, as it consists of two particles bridged by a ~2.7 kbp DNA molecule (Figure 1B, 3A). As shown in the gel image (Figure 1A), no conjugate formation was observed when AuNPs were incubated with either circular pUC19 or the SmaI-linearized blunt-ended plasmid (lanes 2 and 4, respectively). Unlike SmaI, EcoRI generates short 4-base ss overhangs at both ends of the plasmid. These results clearly demonstrate that citrate-protected AuNPs selectively bind to ssDNA fragments and are unable to conjugate with blunt-ended duplex DNA. AFM imaging of the conjugate electroeluted from lane 3 (Figure 1A) clearly shows that the nanoparticles are located exclusively at the DNA termini, with no binding detected along the central regions of the plasmid (Figure 1B), demonstrating that citrate-protected AuNPs bind specifically to single-stranded DNA regions.

### 3.2. DNA Unwinding by Gold Nanoparticle

**Figure 2:**
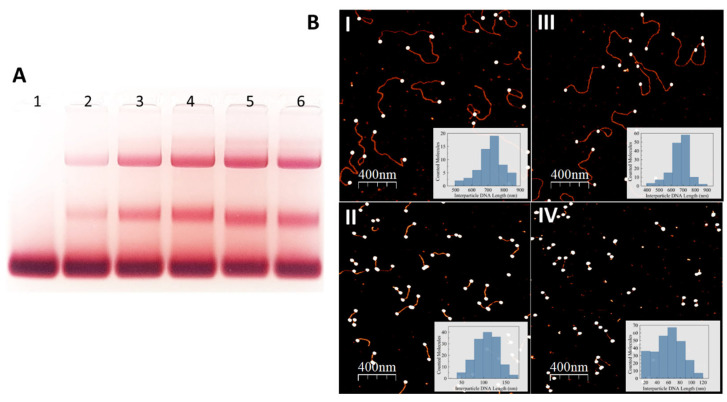
(**A**)—Electrophoresis of conjugates formed between 8 nm AuNPs and pUC19 DNA cleaved with PluTI and AflIII restriction enzymes. The plasmid was linearized as described in Section 2, producing two DNA fragments of 575 bp and 2111 bp, each containing two 4-base ss overhangs. The cleaved DNA was incubated with 8 nm AuNPs (lane 1: AuNPs only) for 30 min (lane 2), 2 h (lane 3), 4 h (lane 4), and 21 h (lane 5) at 4 °C. An additional sample was incubated for 1 h at 25 °C (lane 6). Prior to electrophoresis, 1 mM BSPP and 10% glycerol were added to each sample. The mixtures were electrophoresed on a 3% agarose gel (100 V, 50 min, ambient conditions). DNA-AuNP conjugates were subsequently electroeluted from the gel and analyzed by AFM, as detailed in Section 2. (**B**)—AFM images of the 2111 bp (panels (**I**,**III**)) and 575 bp (panels (**II**,**IV**)) DNA–AuNP conjugates after 30 min and 21 h incubations at 25 °C, respectively. Inserts show statistical contour length distributions. See Table 1 for calculated interparticle lengths values.

To investigate the processes underlying AuNP-DNA interactions, we cleaved an EcoRI-linearized plasmid into two fragments (575 bp and 2111 bp) using the endonucleases PluTI and AflIII. Both fragments contain 4-base ss overhangs at each end, allowing AuNP attachment. Incubation of these fragments with 8 nm AuNPs altered the mobility of the resulting conjugates in agarose gel. The mobility of both conjugates increased with incubation time (compare lanes 2–5 in Figure 2A), indicating progressive shortening of the DNA and a corresponding decrease in the distance between particles within each conjugate. To confirm this, AFM analysis of interparticle distances over the incubation time was performed on AuNP conjugates with PluTI- and AflIII-cleaved pUC19 (Figure 2B). This analysis revealed several key characteristics of the process. First, DNA shortening occurs relatively slowly at low temperatures. At 4 °C, noticeable changes in band mobility and interparticle distances take hours to appear, with almost no change observed during the first 2 h (compare lanes 2 and 3, Figure 2A). In contrast, incubation at 25 °C for 1 h led to a pronounced increase in conjugate mobility (lane 6 in Figure 2A). AFM imaging analysis (Figure 2B, Table 1) showed that the interparticle distance decreased within 30 min by ~120 nm at 25 °C, almost approaching its minimal value. At 4 °C, however, no noticeable change in interparticle distance was observed even after 1 h of incubation.

**Figure 3:**
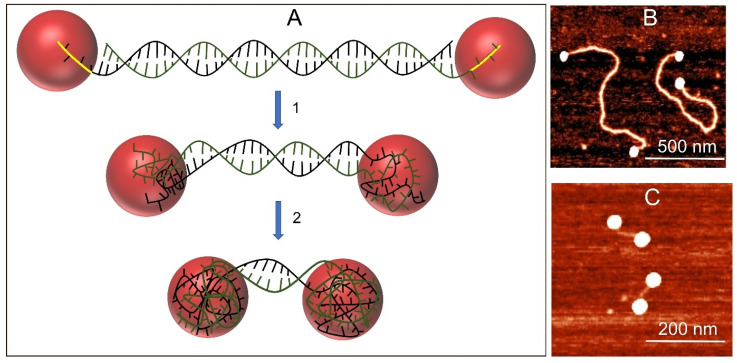
dsDNA unwinding by AuNPs. (**A**) Scheme of the process: AuNPs bind to ss overhangs (yellow curves) at the ends of the duplex and initiate strand separation. As unwinding progresses, the particles (red spheres) become wrapped by the unwound strands (green and black curves), leading to a gradual decrease in the distance between particles within the conjugate. This process is driven by the strong interaction of nucleobases with the nanoparticle surface. (**B**,**C**) AFM images of dumbbells formed following short-term (**B**) and long-term (**C**) incubation of 8 nm AuNPs with linearized plasmid DNA.

These findings suggest that DNA unwinding by AuNPs occurs at comparable rates for both long and short duplexes and continues until the particle surface in the conjugates is completely covered by the resulting ss DNA. A schematic representation of the process is shown in Figure 3. The initial step involves nanoparticle binding to the ssDNA overhangs at both ends of the duplex. Since dsDNA itself cannot bind to the particles, attachment occurs via interactions between the nucleic bases of the overhangs and the AuNP surface. Once bound, the particles begin to unwind the double helix—a process driven by high-affinity base–gold interactions. The reaction is spontaneous, indicating that adsorption of bases onto the gold surface is thermodynamically more favorable than base pairing within the duplex. Unwinding proceeds rather rapidly while the particle surface is largely unoccupied, but gradually slows as more bases adsorb, reducing the number of available binding sites. This reduction in binding capacity decreases the unwinding rate and limits further particle movement along the DNA (Figure S1). Once the particle surface is saturated, the process halts, and the interparticle distance no longer decreases. Indeed, we observed that the final interparticle distance decreases only slightly between 90 min and 7 days of incubation (Table 1). Because DNA unwinding requires overcoming a high activation barrier, the process is strongly temperature dependent. At 4 °C, almost no interparticle distance reduction was observed during 1 h of incubation. The unwinding results in a DNA-AuNP dumbbell, with the interparticle distance equal to the initial DNA length subtracted by the length of the Au-NP-adsorbed segments. In both 2111 bp and 575 bp conjugates with 8 nm AuNP, the interparticle distance decreased by a maximum of ~170 nm, corresponding to approximately 500 bp. As two nanoparticles participate in the process, we can conclude that each 8 nm particle becomes coated with ~500 nucleobases derived from the unwound 250 bp segment.

By varying the dsDNA length and nanoparticle size, the final interparticle distance within the dumbbell structure can be predetermined. Increasing particle size (from 8 nm to 15 nm in this case) is expected to bring the two particles closer. The bigger 15 nm particles conjugated to a short 575 bp plasmid fragment approach each other to a minimal distance, governed by the balance between the closely spaced AuNPs repulsive forces and the thermodynamic drive to continue the DNA unwinding reaction to achieve full surface coverage. The resulting interparticle distance in the dumbbell may even become sufficiently small for plasmon coupling to occur, leading to alterations in the absorption spectrum. Indeed, a pronounced red shift in the absorption spectrum was observed when the 575 bp fragment was incubated with 15 nm AuNPs for an extended period, whereas no shift occurred during short-term incubations (Figure S2A). Prolonged incubation yields DNA-AuNP dumbbells with very short interparticle distances. As shown in the AFM image (Figure S2B), the particles are nearly in contact, accounting for the observed plasmonic shift in the conjugate spectrum (Figure S2A).

To gain further insight into the mechanism of the DNA coating, we quantified the binding capacity of the nanoparticle surface toward nucleic bases by estimating the number of 40-base random-sequence ss that can associate with a 15 nm AuNP. The particles were incubated with a strong (>100-fold) molar excess of strands over particles. The unbound strands were separated from the strand-NP conjugate by chromatography and centrifugation. The spectroscopic analysis of the conjugate (Figure S3A) showed that approximately 23 strands are tightly associated with the particle. Based on these results, we conclude that a single 15 nm AuNP can accommodate approximately 920 DNA bases on its surface. This result fits nicely with the maximal approaching distance achieved during long-term incubation of EcoRI-cleaved pUC19 with 15 nm AuNP (Figure S3B). The distance was reduced by approximately 330 nm, corresponding to a shortening of the double helix by ~970 bp (each base pair contributes ~0.34 nm to the overall length). Using this approach, one can create dumbbells with a desired dsDNA bridge length by selecting the initial dsDNA length, the AuNP diameter and the incubation time and temperature. The particles in the conjugate facilitate the attachment of DNA to a variety of surfaces, including electrodes, enabling conductive measurements of a single DNA molecule. DNA-nanoparticle dumbbells have previously been used to study electrical conductance [14,31], where nanoparticles served as contacts to external electrodes. In earlier designs, however, DNA was typically anchored to nanoparticles via thiol-containing linkers composed of several carbon atoms [14]. These insulating linkers imposed substantial barriers to electron transfer, lowering conductivity and likely contributing to the contradictory results reported in the field [32,33,34,35,36,37,38]. In contrast, the conjugates described here are characterized by strong, stable, and direct attachment of nanoparticles to DNA duplexes spanning tens of bases, thereby eliminating the need for any linkers. This direct connection is expected to enhance electrical conductance and enable more reliable and reproducible measurements by facilitating efficient electron injection directly into the DNA. Thus, this system can be used to probe the properties of surface-deposited DNA duplexes; the dumbbells can also act as modular building blocks in complex DNA–nanoparticle architectures and serve as versatile components for designing nanoscale devices with tailored structural and electronic properties.

Our results demonstrate that AuNPs are capable of unwinding DNA duplexes. In cells, DNA unwinding is catalyzed by helicases, enzymes, which act at the replication fork and use energy from ATP hydrolysis to separate the two complementary DNA strands. By analogy, one can envision the nanoparticles as mimicking the action of these enzymes. We are aware that this analogy is speculative and the cellular process differs significantly from what we observe in our experiments. A key distinction is that, in enzyme-catalyzed unwinding, the released ssDNA strands remain in solution and are stabilized by ssDNA-binding proteins. In contrast, during nanoparticle-driven unwinding, the separated strands remain tightly adsorbed to the nanoparticle surface. The only way to release them is to completely disintegrate the nanoparticles into gold atoms. This can be achieved by treating the conjugates with potassium cyanide (KCN), which completely dissolves the particles, yielding [Au(CN)_2_]^−^ [39].

The treatment led to the removal of nanoparticles from the conjugates, as observed in AFM images (Figure S4). The bright spots at the ends of DNA fibers corresponding to AuNPs (Figure S4A) disappear after the KCN treatment (Figure S4B). After particle dissolution, the unwound DNA strands that were previously captured by the particles do not reassemble into the original DNA. Small grains and irregular fragments at the DNA ends can be clearly seen in AFM images of the CN-treated conjugates (Figure S4B, indicated by white arrows). These results suggest that rapid strands hybridization following fast disintegration of AuNPs by CN^−^ does not always yield a complete double helix. In some cases, fragments of individual strands can fold into relatively stable intramolecular secondary structures, such as hairpins, which makes hybridization with their complementary strands less likely.

## Figures and Tables

**Table 1:** Interparticle distance * at different incubation conditions.

Incubation Time	Temperature (°C)	AuNP Diameter (nm)	Interparticle DNA Length (nm)		Counted Molecules	
			575 bp. Fragment	2111 bp. Fragment	575 bp. Fragment	2111 bp. Fragment
0		8	221 ± 32	825 ± 113	221	143
30 min	25	8	107 ± 25	713 ± 80	146	57
90 min	25	8	90 ± 22	676 ± 66	221	113
7 days	25	8	59 ± 24	665 ± 77	274	144
7 days	25	15	30 (no DNA seen between NPs)	448 ± 70	70	173

* Distances were measured as shown in Figure 2.

## Data Availability

All data are provided in the paper.

## Supplementary Materials

The following supporting information can be downloaded at: https://www.mdpi.com/article/10.3390/nano15241872/s1, Figure S1. Time-dependent changes in the length of DNA bridging the particles in the dumbbells. Figure S2. Absorption spectroscopy (A) and AFM imaging (B) of a 575 bp pUC19 fragment incubated with 15 nm AuNPs for 16 h at 25 °C. Figure S3. AuNPs binding capacity for DNA nucleotides. Figure S4. AFM analysis of DNA in a cyanide-treated conjugate of 15 nm AuNPs with EcoRI-cleaved pUC19.

## References

[1] Giljohann D.A., Seferos D.S., Daniel W.L., Massich M.D., Patel P.C., Mirkin C.A. (2010). Gold Nanoparticles for Biology and Medicine. Angew. Chem. Int. Ed. Engl..

[2] Dreaden E.C., Alkilany A.M., Huang X., Murphy C.J., El-Sayed M.A. (2012). The Golden Age: Gold Nanoparticles for Biomedicine. Chem. Soc. Rev..

[3] Dutour R., Bruylants G. (2025). Gold Nanoparticles Coated with Nucleic Acids: An Overview of the Different Bioconjugation Pathways. Bioconjug. Chem..

[4] Wang C.C., Wu S.M., Li H.W., Chang H.T. (2016). Biomedical Applications of DNA-Conjugated Gold Nanoparticles. ChemBioChem.

[5] Niemeyer C.M. (2001). Nanoparticles, Proteins, and Nucleic Acids: Biotechnology Meets Materials Science. Angew. Chem..

[6] Lazarides A.A., Schatz G.C. (2000). DNA-Linked Metal Nanosphere Materials: Structural Basis for the Optical Properties. J. Phys. Chem. B.

[7] Thacker V.V., Herrmann L.O., Sigle D.O., Zhang T., Liedl T., Baumberg J.J., Keyser U.F. (2014). DNA Origami Based Assembly of Gold Nanoparticle Dimers for Surface-Enhanced Raman Scattering. Nat. Commun..

[8] Piantanida L., Naumenko D., Lazzarino M. (2014). Highly Efficient Gold Nanoparticle Dimer Formation via DNA Hybridization. RSC Adv..

[9] Borovok N., Gillon E., Kotlyar A. (2012). Synthesis and Assembly of Conjugates Bearing Specific Numbers of Dna Strands per Gold Nanoparticle. Bioconjug. Chem..

[10] Busson M.P., Rolly B., Stout B., Bonod N., Larquet E., Polman A., Bidault S. (2011). Optical and Topological Characterization of Gold Nanoparticle Dimers Linked by a Single DNA Double Strand. Nano Lett..

[11] Samanta D., Zhou W., Ebrahimi S.B., Petrosko S.H., Mirkin C.A. (2022). Programmable Matter: The Nanoparticle Atom and DNA Bond. Adv. Mater..

[12] Yao H., Yi C., Tzang C.H., Zhu J., Yang M. (2007). DNA-Directed Self-Assembly of Gold Nanoparticles into Binary and Ternary Nanostructures. Nanotechnology.

[13] Maye M.M., Lim I.I.S., Luo J., Rab Z., Rabinovich D., Liu T., Zhong C.J. (2005). Mediator-Template Assembly of Nanoparticles. J. Am. Chem. Soc..

[14] Zhuravel R., Stern A., Fardian-Melamed N., Eidelshtein G., Katrivas L., Rotem D., Kotlyar A.B., Porath D. (2018). Advances in Synthesis and Measurement of Charge Transport in DNA-Based Derivatives. Adv. Mater..

[15] Heintz J., Markešević N., Gayet E.Y., Bonod N., Bidault S. (2021). Few-Molecule Strong Coupling with Dimers of Plasmonic Nanoparticles Assembled on DNA. ACS Nano.

[16] Halamish S., Eidelshtein G., Kotlyar A. (2013). Plasmon-Coupled Nanostructures Comprising Finite Number of Gold Particles. Plasmonics.

[17] Seow N., Tan Y.N., Yung L.Y.L., Su X. (2015). DNA-Directed Assembly of Nanogold Dimers: A Unique Dynamic Light Scattering Sensing Probe for Transcription Factor Detection. Sci. Rep..

[18] Hutter E., Maysinger D. (2013). Gold-Nanoparticle-Based Biosensors for Detection of Enzyme Activity. Trends Pharmacol. Sci..

[19] Templeton A.C., Wuelfing W.P., Murray R.W. (2000). Monolayer-Protected Cluster Molecules. Acc. Chem. Res..

[20] Liu B., Liu J. (2017). Methods for Preparing DNA-Functionalized Gold Nanoparticles, a Key Reagent of Bioanalytical Chemistry. Anal. Methods.

[21] Kimura-Suda H., Petrovykh D.Y., Tarlov M.J., Whitman L.J. (2003). Base-Dependent Competitive Adsorption of Single-Stranded DNA on Gold. J. Am. Chem. Soc..

[22] Irrera S., Portalone G., De Leeuw N.H. (2013). Chemisorption of Uracil on Gold Surfaces via Density Functional Theory. Surf. Sci..

[23] Nelson E.M., Rothberg L.J. (2011). Kinetics and Mechanism of Single-Stranded DNA Adsorption onto Citrate-Stabilized Gold Nanoparticles in Colloidal Solution. Langmuir.

[24] Zhang X., Servos M.R., Liu J. (2012). Surface Science of DNA Adsorption onto Citrate-Capped Gold Nanoparticles. Langmuir.

[25] Carnerero J.M., Jimenez-Ruiz A., Castillo P.M., Prado-Gotor R. (2017). Covalent and Non-Covalent DNA–Gold-Nanoparticle Interactions: New Avenues of Research. ChemPhysChem.

[26] Cárdenas M., Barauskas J., Schullén K., Brennan J.L., Brust M., Nylander T. (2006). Thiol-Specific and Nonspecific Interactions between DNA and Gold Nanoparticles. Langmuir.

[27] Sandström P., Boncheva M., Åkerman B. (2003). Nonspecific and Thiol-Specific Binding of DNA to Gold Nanoparticles. Langmuir.

[28] FRENS G. (1973). Controlled Nucleation for the Regulation of the Particle Size in Monodisperse Gold Suspensions. Nat. Phys. Sci..

[29] Dong J., Carpinone P.L., Pyrgiotakis G., Demokritou P., Moudgil B.M. (2020). Synthesis of Precision Gold Nanoparticles Using Turkevich Method. KONA Powder Part. J..

[30] Horcas I., Fernández R., Gómez-Rodríguez J.M., Colchero J., Gómez-Herrero J., Baro A.M. (2007). WSXM: A Software for Scanning Probe Microscopy and a Tool for Nanotechnology. Rev. Sci. Instrum..

[31] Park S.J., Lazarides A.A., Mirkin C.A., Brazis P.W., Kannewurf C.R., Letsinger R.L. (2000). The Electrical Properties of Gold Nanoparticle Assemblies Linked by DNA. Angew. Chem..

[32] Porath D., Bezryadin A., De Vries S., Dekker C. (2000). Direct Measurement of Electrical Transport through DNA Molecules. Nature.

[33] Fink H.W., Schönenberger C. (1999). Electrical Conduction through DNA Molecules. Nature.

[34] Xu B., Zhang P., Li X., Tao N. (2004). Direct Conductance Measurement of Single DNA Molecules in Aqueous Solution. Nano Lett..

[35] Kasumov A.Y., Kociak M., Guéron S., Reulet B., Volkov V.T., Klinov D.V., Bouchiat H. (2001). Proximity-Induced Superconductivity in DNA. Science.

[36] Dekker G., Ratner M.A. (2001). Electronic Properties of DNA. Phys. World.

[37] Okahata Y., Kobayashi T., Tanaka K., Shimomura M. (1998). Anisotropic Electric Conductivity in an Aligned DNA Cast Film. J. Am. Chem. Soc..

[38] Giese B., Amaudrut J., Köhler A.K., Spormann M., Wessely S. (2001). Direct Observation of Hole Transfer through DNA by Hopping between Adenine Bases and by Tunnelling. Nature.

[39] Biellmann J.F., Jung M.J. (1970). Preparation and Properties of 3-Cyano Pyridine Ad+, a New Analogue of Nad+. FEBS Lett..

