# Supplementary material for: DNA Unwinding Driven by Gold Nanoparticles
Source: Nanomaterials (Basel). 2025 Dec 13;15(24):1872. doi: 10.3390/nano15241872 (PMC12735733; doi:10.3390/nano15241872)
Supplement: Supplementary file 1 [file nanomaterials-15-01872-s001.zip › nanomaterials-3974235-supplementary.pdf]

Supporting Information

DNA Unwinding Driven by Gold Nanoparticles

Liat Katrivas [1], Galina M. Proshkina [2], Sergey M. Deyev [2] and Alexander B. Kotlyar [1,*]

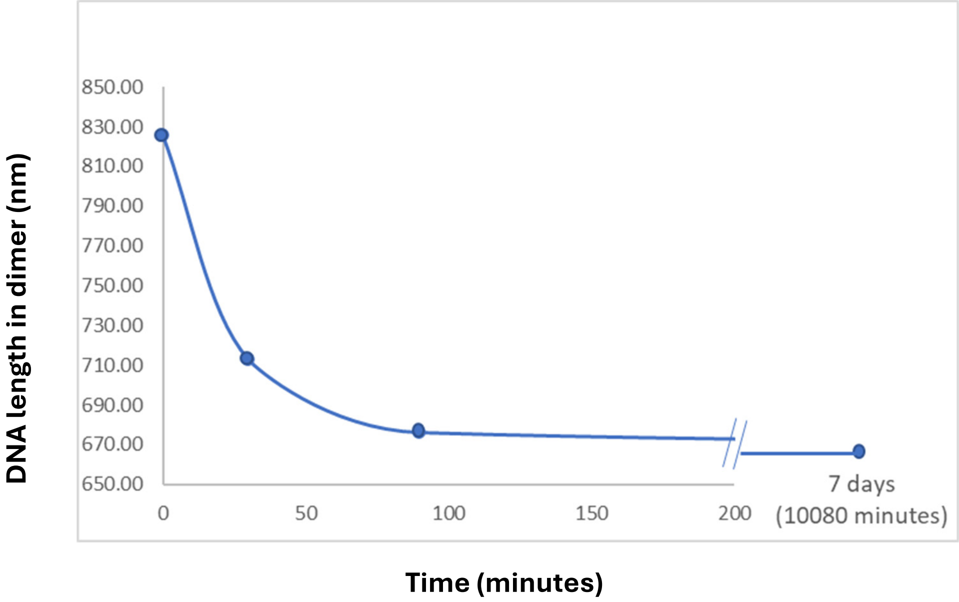

Figure S1. Time-dependent changes in the length of DNA bridging the particles in the dumbbells. The dumbbells were formed by incubating 8 nm AuNPs with a 2111 bp DNA fragment derived from pUC19. DNA lengths were measured at 0, 30, and 90 minutes, as well as after 7 days incubation at 25 °C. More than 100 dumbbells were analyzed at each time point. The corresponding average inter-particle lengths were: 825 ± 113, 713 ± 80, 676 ± 66, and 665 ± 77 nm.

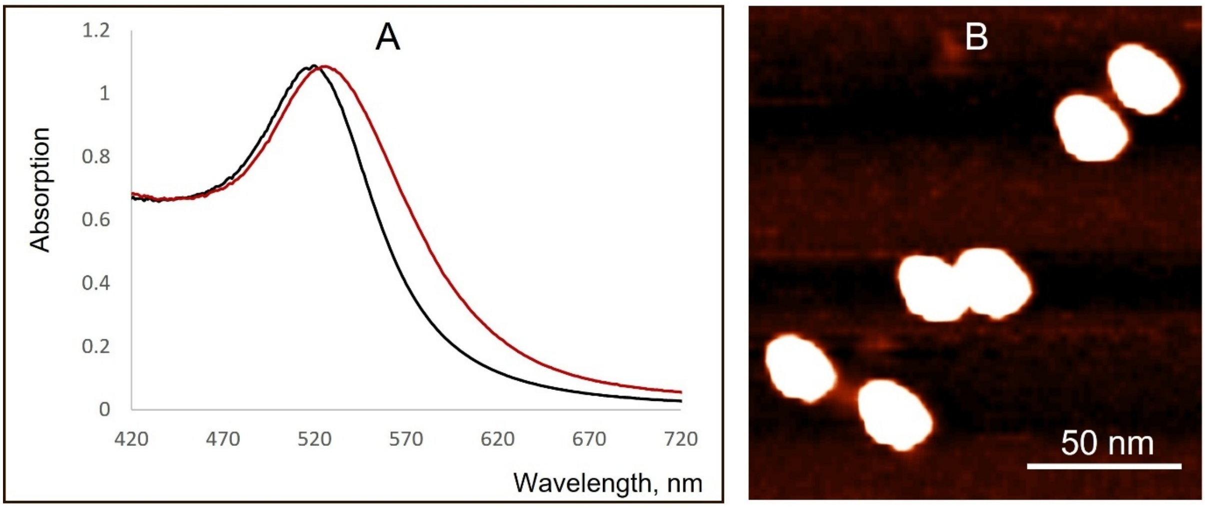

Figure S2. Absorption spectroscopy (A) and AFM imaging (B) of a 575 bp pUC19 fragment incubated with 15 nm AuNPs for 16 hours at 25 °C. A - The particles were incubated with DNA for 10 minutes (black curve) and 16 hours at 25 °C (red curve). The 16-hour conjugate was electrophoresed, electroeluted from the gel and imaged by AFM as described in Section 2.

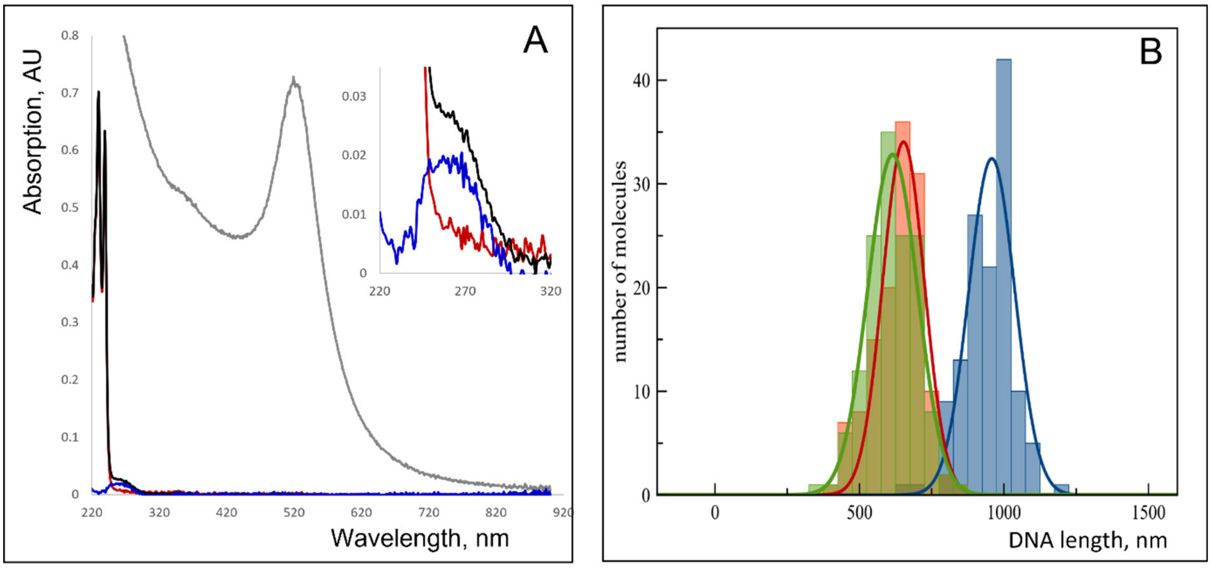

Figure S3. AuNPs binding capacity for DNA nucleotides. (A) 40-base random ssDNA (see sequence in Section 2) was conjugated to 15 nm AuNPs, and the resulting conjugate was purified as described in Section 2. The absorption spectrum of the conjugate is shown in grey. The conjugate was then treated with KCN for 30 minutes, to dissolve the AuNPs, forming the $Au(CN)_2^-$ complex (absorption spectrum shown in black). The two sharp peaks in the black curve correspond to the absorption maxima of $Au(CN)_2^-$. A small broad peak with maximum at 260 nm (black curve; inset) corresponds to the oligonucleotide released into solution upon particle dissolution. By subtracting the contribution of $Au(CN)_2^-$ (red curve) from the spectrum of the dissolved conjugate (black curve), the spectrum of the liberated oligonucleotide was obtained (blue curve; inset). The oligonucleotide concentration was calculated using an extinction coefficient of 3.8×10^5 $M^{-1}{\cdot}cm^{-1}$ at 260 nm. The concentration of 15 nm AuNPs was determined using an extinction coefficient of 3×10^8 $M^{-1}cm^{-1}$ at 525 nm. Therefore, the estimated concentrations of oligonucleotide and AuNPs were 53 nM and 2.3 nM, respectively, giving an oligonucleotide-to-particle ratio of 53 nM / 2.3 nM ≈ 23. Accordingly, a single 15 nm AuNP can accommodate about 920 DNA bases (23 × 40). B – 15 nm AuNPs were incubated with EcoRI-cleaved pUC19 DNA for 16 hours at 4 °C (blue), 16 hours at 37 °C (red), and 90 hours at 37 °C (green). The resulting conjugates were electrophoresed, electroeluted from the gel, and imaged by AFM as described in Section 2. More than 130 DNA dumbbells were analyzed at each time point. The corresponding average lengths measured by AFM were 923 ± 83, 607 ± 83, 587 ± 84. Solid lines indicate Gaussian fits to the data.

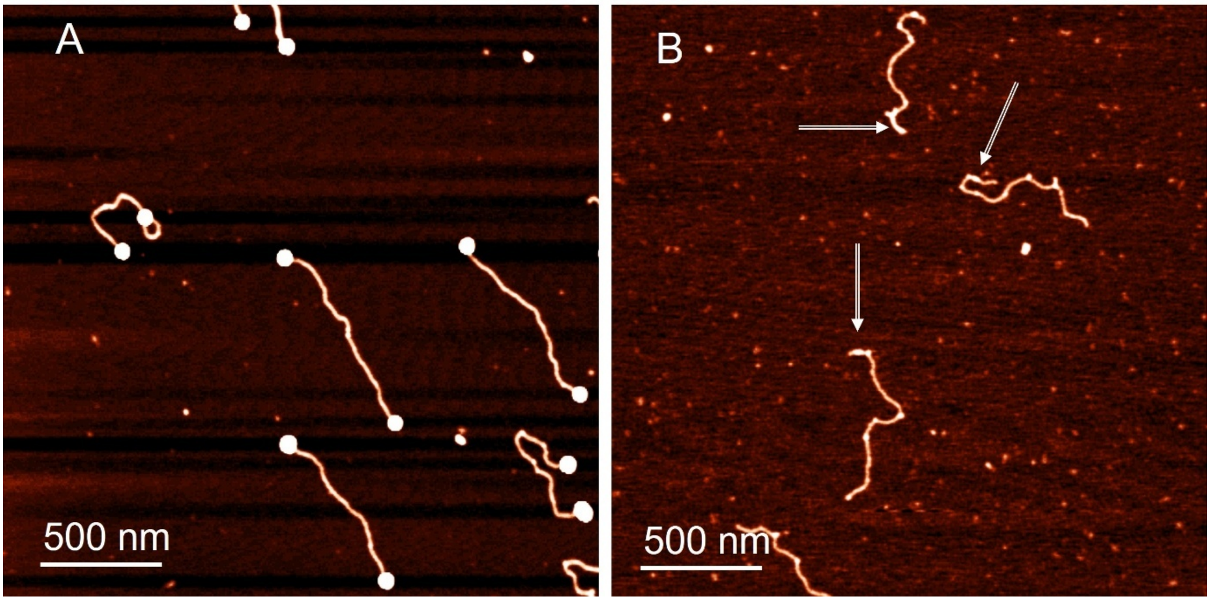

Figure S4. AFM analysis of DNA in a cyanide-treated conjugate of 15 nm AuNPs with EcoRI-cleaved pUC19. The conjugate was prepared by incubating 15 nm AuNPs with EcoRI-cleaved pUC19 for 16 hours at 25 °C, as described in Section 2. Conjugate purification by electrophoresis, elution from the gel, and AFM imaging were carried out as described in Section 2. AFM images of the conjugate before and after cyanide treatment are shown in panels A and B, respectively. White arrows in panel B indicate irregular regions in the DNA.
